# Day 7 Embryos Change the Proteomics and Exosomal Micro-RNAs Content of Bovine Uterine Fluid: Involvement of Innate Immune Functions

**DOI:** 10.3389/fgene.2021.676791

**Published:** 2021-06-28

**Authors:** Kazuya Kusama, Mohammad B. Rashid, Rasoul Kowsar, Mohamed A. Marey, Anup K. Talukder, Kentaro Nagaoka, Masayuki Shimada, Hasan Khatib, Kazuhiko Imakawa, Akio Miyamoto

**Affiliations:** ^1^Department of Endocrine Pharmacology, Tokyo University of Pharmacy and Life Sciences, Tokyo, Japan; ^2^Global Agromedicine Research Center (GAMRC), Obihiro University of Agriculture and Veterinary Medicine, Obihiro, Japan; ^3^Department of Physiology and Pharmacology, Hajee Mohammad Danesh Science and Technology University, Dinajpur, Bangladesh; ^4^Department of Animal Sciences, College of Agriculture, Isfahan University of Technology, Isfahan, Iran; ^5^Department of Theriogenology, Faculty of Veterinary Medicine, Damanhour University, Damanhour, Egypt; ^6^Department of Gynecology, Obstetrics and Reproductive Health, Bangabandhu Sheikh Mujibur Rahman Agricultural University, Gazipur, Bangladesh; ^7^Laboratory of Veterinary Physiology, Cooperative Department of Veterinary Medicine, Faculty of Agriculture, Tokyo University of Agriculture and Technology, Tokyo, Japan; ^8^Graduate School of Integrated Sciences for Life, Hiroshima University, Higashi-Hiroshima, Japan; ^9^Department of Animal and Dairy Sciences, University of Wisconsin-Madison, Madison, WI, United States; ^10^Research Institute of Agriculture, Tokai University, Kumamoto, Japan

**Keywords:** Day 7 embryo, uterine flushing, protein, exosome, miRNA, immunity, cow

## Abstract

This study aimed to characterize proteins and exosomal microRNAs (miRNAs) in the uterine flushings (UF) of cows associated with Day 7 (D7) pregnancy using the embryo donor cows of the embryo transfer program. Superovulated cows either were inseminated (AI cows) or remained non-inseminated (Ctrl cows). UF was collected on D7 in the presence of multiple embryos (AI cows) or without embryos (Ctrl cows) and subjected to isobaric tags for relative and absolute quantification protein analysis. A total of 336 proteins were identified, of which 260 proteins were more than 2-fold higher in AI cows than Ctrl cows. Gene ontology analysis revealed that many differentially expressed proteins were involved in “neutrophil-related” and “extracellular vesicular exosome-related” terms. In silico analysis of proteins with higher concentrations in the UF of AI identified 18 uniquely expressed proteins. Exosomes were isolated from the UF, from which RNA was subjected to miRNA-seq, identifying 37 miRNAs. Of these, three miRNAs were lower, and six miRNAs were higher in the UF of AI cows than those of Ctrl ones. The principal component analysis displayed a close association in miRNA and protein between bta-miR-29a, bta-miR-199b, SUGT1, and PPID. In addition, the receiver operating characteristic curve analysis showed that SUGT1 was the best predictor for the presence of embryos in the uterus. These findings suggest that the presence of multiple D7 embryos in the uterus can lead to significant changes in the protein composition and exosomal miRNA contents of UF, which could mediate innate immunological interactions between the pre-hatching embryo and the uterus in cows.

## Introduction

Up to 50% of potential bovine pregnancies are lost in the first week following insemination (Diskin and Morris, 2008; Wiltbank et al., 2016). Thus, proper interactions between the early embryo and mother are crucial for successful pregnancy in cows. Pregnancy is established by complex interactions of the factors/molecules derived from both the developing embryo and the cow’s endometrium (Talukder et al., 2020). The pre-hatching bovine blastocyst can synthesize and secrete interferon-tau (IFNT), the pregnancy recognition signal in ruminants, on Day 7 (D7) of pregnancy. Subsequently, IFNT regulates the maternal immune response toward an anti-inflammatory (Th2) action to tolerate the semi-allogenic embryo (Sponchiado et al., 2017; Talukder et al., 2017; Passaro et al., 2018; Rashid et al., 2018; Sponchiado et al., 2019). In addition to IFNT, other factors from both the pre-hatching embryo or the endometrium may modulate maternal immunity. However, the nature of these interactions during the pre-hatching period of pregnancy has not yet been well characterized.

Several global analyses of bovine uterine flushing (UF) revealed some changes in intrauterine protein levels during the peri-implantation period (Forde et al., 2013, 2014a,b; Kusama et al., 2016, 2018b). Furthermore, extracellular vesicles (EVs), including exosomes, have been identified in UF (Burns et al., 2014; Nakamura et al., 2016; Kusama et al., 2018b) and found to be involved in the conceptus-endometrial interactions during the implantation period (Burns et al., 2016; Nakamura et al., 2016; Kusama et al., 2018b). In sheep, endometrial exosomes regulate IFNT production by the conceptus (Ruiz-González et al., 2015). Several studies investigated the effects of intrauterine exosomes on immunological interactions between the conceptus and endometrium during the peri-implantation period (Burns et al., 2014, 2016; Nakamura et al., 2016; Kusama et al., 2018b), but not in earlier stages, particularly during the pre-hatching period in cows.

MicroRNAs (miRNAs) are small, non-coding RNAs that regulate gene expression in various cell types (Bi et al., 2009). The pre-hatching D7 bovine blastocysts can secrete miRNAs into the extracellular environment through EVs/exosomes (Gross et al., 2017b). Given that both the early embryo and the maternal tract produce miRNAs, these miRNAs may serve as communication signals between the pre-hatching embryo and mother during early pregnancy (Gross et al., 2017b). miRNAs were found to be involved in gametogenesis, early embryo development, implantation, and placental formation (Bidarimath et al., 2014; Ioannidis and Donadeu, 2016; Gross et al., 2017a). Recent studies also indicate that miRNAs contribute to the immune tolerance at conception through regulatory effects of seminal fluid in generating tolerogenic dendritic cells and T-reg cells (Bidarimath et al., 2014; Schjenken et al., 2016).

We hypothesized that the presence of D7 embryos could induce global changes in the protein composition and exosomal contents of UF during the pre-hatching period, regulating the local uterine immunity on D7 post-insemination for the embryo tolerance, thereby ensuring the establishment of pregnancy in cows. Therefore, the present study aimed to investigate the embryo- and endometrium-derived factors involved in regulating the local immune environment of the uterus at D7 of pregnancy in cows. Additionally, bioinformatics analysis was used to identify significant proteins and miRNAs and their interrelationship associated with D7 pregnancy in cows. In the present investigation, we used a superovulation model for multiple embryo production in embryo transfer donor cows. This model could amplify embryo-derived signals in the uterus that can not be easily detected using the single-embryo model. Indeed, we previously found that interferon-stimulated genes were upregulated in circulating immune cells in the superovulation cow model (Talukder et al., 2019). Furthermore, we used the Japanese Black cows that were prepared exclusively for embryo donors and have only one-time experience of pregnancy and lactation, so their responsiveness to hormone treatment was expected to be highly homogeneous.

## Materials and Methods

### Ethics Statement

All animal experiments were conducted in a commercial herd (Nobel’s Co. Ltd., Hokkaido, Japan) under the approval of the Animal Experiments Ethics Committee, Obihiro University of Agriculture and Veterinary Medicine, Japan (Permit number 25–101).

### Experimental Design

**Figure 1:**
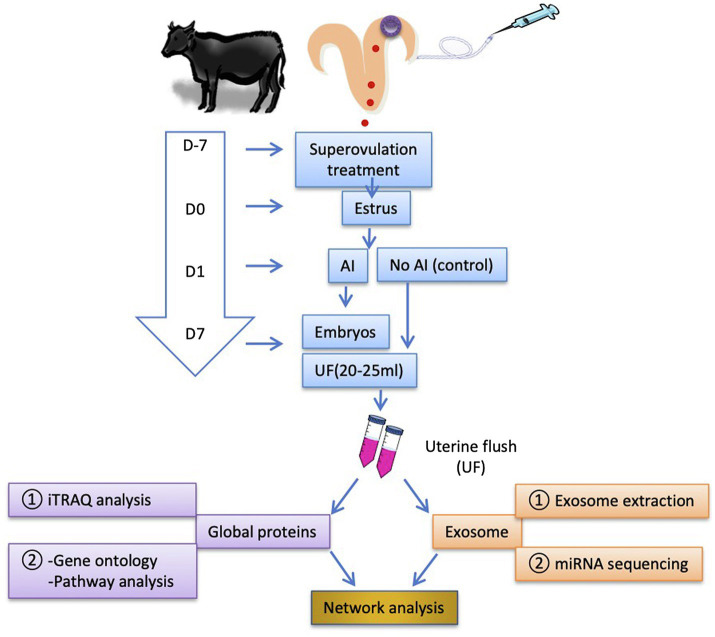
Schematic illustration of the experimental design. Cows were superovulated 7 days before the estrus (D7). In estrus (D0), the cows were either inseminated (AI cows) or non-inseminated (Ctrl cows) on D1. The embryos were flushed out on D7, and the first uterine flush (UF) was collected (20–25 ml). To reduce individual variations in the animal responses and the cost for analyses, the equal volume of UF samples was pooled together (UF from each three AI cows and each two Ctrl cows were pooled as a single sample, respectively; thus, three pooled AI UF from nine cows and three pooled Ctrl UF from six cows) and stored at −80°C until use. The number of recovered embryos with good quality per AI cow was 9.5 ± 1.4 (mean ± SEM). Global UF protein analysis was performed using isobaric tags for relative and absolute quantification (iTRAQ) analysis followed by GO and enriched signaling pathway analyses. Exosomes were isolated from D7 UF followed by RNA extraction and microRNA (miRNA) sequencing for the identification of exosome-derived miRNAs contents. The identified proteins and miRNAs were mapped to network analysis. AI, artificial insemination; UF, uterine flush; and D, day.

To investigate the effect of multiple D7 embryos on uterine proteomic and exosomal miRNA contents, donor Japanese Black cows (3–7 years old) were treated for superovulation 7 days before estrus (D7). UF (20–25 ml) was collected on D7 from inseminated cows and remained non-inseminated cows on D0 (estrus). Pregnancy was confirmed by the presence of multiple embryos in the UF. The UF content was analyzed using isobaric tags for relative and absolute quantification (iTRAQ) analysis, followed by GO and enriched signaling pathway analyses. Exosomes were isolated from the UF, followed by RNA extraction and miRNA sequencing. Proteins and miRNAs were subjected to network analysis to identify associations with D7 pregnancy outcomes in cows (Figure 1).

### Generation and Collection of D7 Bovine Uterine Flushing

Twenty-one cows from a commercial herd (Nobel’s Co. Ltd., Hokkaido, Japan) were assigned randomly to the experiment. All cows were synchronized for estrus using the superovulation regimen as described previously (Rashid et al., 2018). Based on the behavioral estrus on D0, cows were either inseminated (*n* = 15) or remained non-inseminated (*n* = 6). On D7, embryos were non-surgically flushed out from the uterus using Ringer solution (SOLULACT, Terumo Co., Tokyo, Japan, 500 ml/uterine horn × 2 horns) as described in Rashid et al. (2018). Briefly, a balloon catheter (Fujihira Industries Co., Tokyo, Japan) was first inserted up to the tip of the uterine horn ipsilateral to the side existing a higher number of corpora lutea in the ovary, and an inflatable cuff on the catheter was filled with air to hold the catheter in place. Flushing solution (20–25 ml) was infused inside the uterus and rapidly aspirated to avoid fluid overflow inside the lumen of the uterus and prevent injury of the soft uterine horn. The flushing process was repeated several times using small amounts of Ringer solution (totally 500 ml/uterine horn × 2 horns). Embryos were classified and graded according to the guidelines of the International Embryo Transfer Society (Bó and Mapletoft, 2014). Only inseminated cows, referred to as AI cows (*n* = 9), with relatively large numbers of good quality embryos (Grades 1 and 2) were used in this experiment. The first 20–25 ml of UF collected from the uterine horn ipsilateral to the side existing a higher number of corpora lutea in the ovary was kept for further analysis. The Ctrl cows were exposed to a single uterine flushing process using 20–25 ml of UF. To minimize individual variation in the animal responses, three pools of UF samples from AI cows and three pools from Ctrl cows were constructed. Each pool comprised an equal volume (4 ml) of UF. Pooled samples were centrifuged at 1,000 × g for 15 min, and the supernatant was collected and stored at −80°C until use.

### iTRAQ Analysis

Global protein analysis was performed using iTRAQ analysis as described previously (Kusama et al., 2018b). Briefly, total protein (100 μg) from D7 UF samples was subjected to trypsin digestion and then reacted with appropriate iTRAQ reagent according to the manufacturer’s instructions. Sample fractionation was performed with an Agilent 3100 OFFGEL Fractionator (Agilent Technologies, Santa Clara, CA, United States). Mass spectrometry analysis was performed with a Thermo Scientific LTQ Orbitrap XL mass spectrometer (Thermo Fisher Scientific, Waltham, MA, United States). Mascot software was used to identify and quantify proteins simultaneously.

### Isolation of Exosomes From the Uterine Flush

Exosomes were isolated from D7 UF using exosome precipitation solution (Exo-Quick-TC, System Biosciences, Mountain View, CA, United States) according to the manufacturer’s instructions. The UF with Exo-Quick-TC was incubated overnight at 4°C and then centrifuged at 1,500 × g for 30 min at 4°C to pellet exosomes as previously described (Nakamura et al., 2016; Kusama et al., 2018b).

### Western Blotting

Exosomes lysed with M-PER (10 μg) were separated through SDS-PAGE, then transferred onto polyvinylidene difluoride membranes (Bio-Rad, Hercules, CA, United States). After blocking with bullet blocking one (Nacalai Tesque, Kyoto, Japan), the membranes were incubated with rabbit polyclonal anti-AKR1B1 (1:500, sc-33219, Santa Cruz Biotechnology, Dallas, TX, United States), goat polyclonal anti-CAPG (1:100, sc-33084, Santa Cruz Biotechnology), rabbit polyclonal anti-HSP70 antibody (1:2,000, EXOAB-HSP70A-1, System Biosciences), rabbit polyclonal anti-CD63 (1:1,000, EXOAB-CD63A-1, System Biosciences), or rabbit polyclonal anti-RAB5 (ab13253, 1:1,000, Abcam, Tokyo, Japan). Immunoreactive bands were detected using enhanced chemiluminescence (EMD Millipore, Temecula, CA, United States) after incubation with horseradish peroxidase-labeled goat anti-rabbit IgG or horse anti-goat IgG (1:5,000, Vector Laboratories, Burlingame, CA, United States). Signals were detected using C-DiGit Blot Scanner (LI-COR; Kusama et al., 2018a). Total proteins were stained with colloidal gold total protein stain solution according to the manufacturer’s instructions (Bio-Rad Laboratories, Hercules, CA, United States).

### Nanoparticle Tracking Analysis

Nanoparticle tracking analysis of exosomes, isolated from D7 UF and suspended in PBS (2–6 × 10^8^ particles/ml) was performed using a NanoSight NS300 (NanoSight Ltd., Amesbury, United Kingdom) instrument with 488 nm laser and a complementary metal oxide-semiconductor camera (Andor Technology, Belfast, United Kingdom) and NanoSight NTA 3.2 software calibrated with 100 nm polystyrene beads (Thermo Fisher Scientific; Kusama et al., 2017).

### RNA Extraction and miRNA Sequencing

For miRNA-seq analysis, RNA was extracted from exosomes using the SeraMir Exosome RNA Amplification Kit (System Biosciences) according to the manufacturer’s instructions. The miRNA libraries were constructed using the SMARTer smRNA-seq kit (Clontech, Tokyo, Japan) according to the manufacturer’s instructions. RNA sequencing was performed on an Illumina Hiseq 2500 platform (Macrogen, Tokyo, Japan), and 50-bp, single-end reads were generated. Trimmed reads of 18–50 nt were mapped to a reference sequence by Bowtie. Mapped fragments were annotated with a known miRNA database (miRBase22.1) using feature counts. Annotated data were normalized using edgeR to the total reads of each sample as the standardized to count per million. The primary data were deposited to the (DNA Data Bank of Japan) Sequence Read Archive^1^ (accession number DRA010067). Data analysis was performed as described previously (Nakamura et al., 2020).

### Statistical Analysis

Data are expressed as the mean ± SEM. Significance was assessed using ANOVA and the Dunnet comparisons test. A value of *p* < 0.05 was considered statistically significant. Gene ontology (GO) and enriched signaling pathway analyses were performed with the Enrichr tool.^2^ The multivariate method, the principal component analysis (PCA), was used to estimate the association of the inter-correlated variables (i.e., an association between proteins and the presence of embryos in the UF or an association between proteins and miRNAs). Network analysis with the Pearson similarity index was performed to identify central nodes (i.e., proteins or miRNAs). The Fruchterman-Reingold algorithm was used as a force-directed layout algorithm. For PCA and network mapping, the PAST program (accessible at http://folk.uio.no/ohammer/past) was used. Using the easyROC web-tool,^3^ a receiver operating characteristic (ROC) curve analysis was performed to identify predictive factors for the presence of embryos. The optimal cutoff for the presence of embryos was determined by maximizing the Youden index. Finally, the area under the curve (AUC) was determined by the asymptotic statistic (a parametric unbiased likelihood estimator) to detect UF-containing embryos.

## Results

### Quantitative Changes in the Protein Abundance of D7 UF

**Figure 2:**
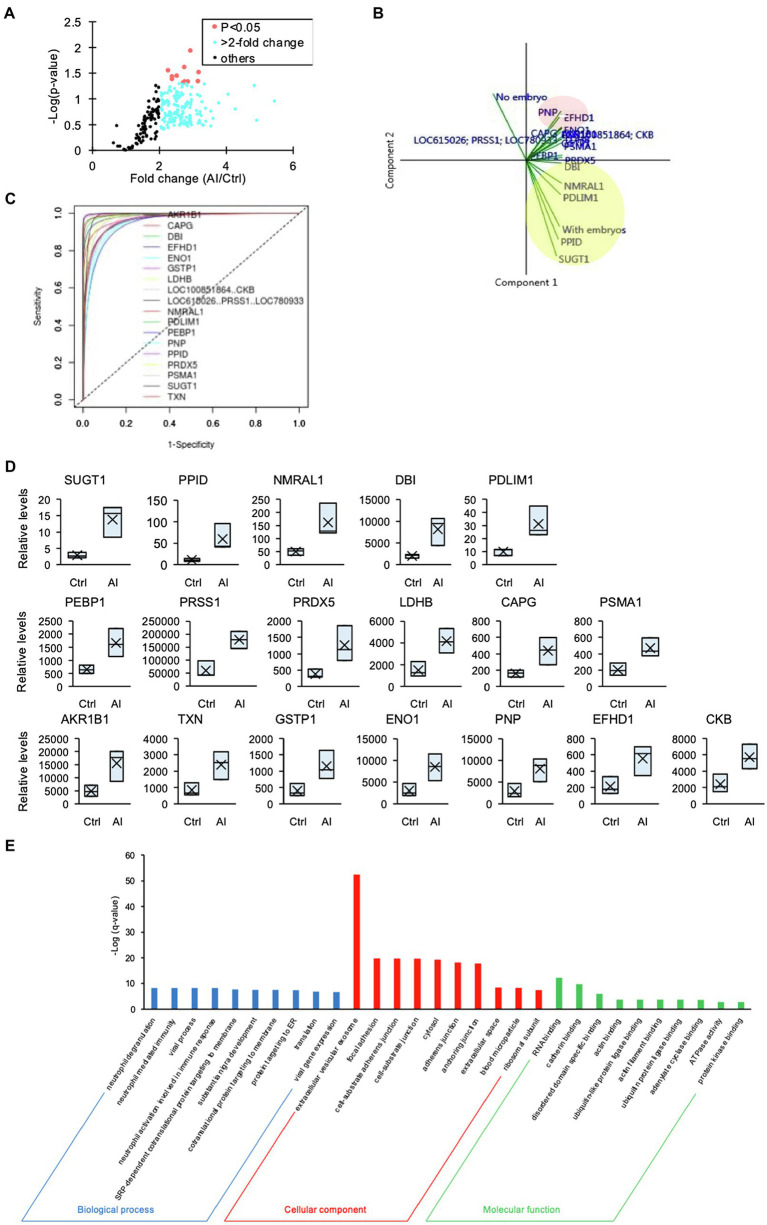
The iTRAQ analysis of uterine flush (UF) collected on D7. **(A)** Volcano plot showing the protein levels found by the iTRAQ analysis. The proteins highlighted in light blue are more than 2-fold higher, and those proteins highlighted in red are significantly higher (*p* < 0.05) in UF-containing embryos obtained from inseminated (AI) cows. **(B)** Biplot produced by the principal component analysis (PCA) of factors, including higher-concentration proteins in UF of cows. Close-angles vectors (<45°) indicate a strong correlation, perpendicular vectors indicate no correlation, and vectors in opposite directions (approaching 180°) indicate a negative correlation. **(C)** Receiver operating characteristic (ROC) curve analysis was performed to assess the predictive power of variables and to measure the optimum cutoff point for the presence of embryos in UF. **(D)** Changes in identified proteins by the comparison between Ctrl and AI cows were determined by iTRAQ analysis. Upper box plots were AUC > 0.99 by ROC. **(E)** Higher-concentration proteins in UF of AI cows were functionally classified in GO analysis by the biological process (BP), cellular component (CC), and molecular function (MF) terms.

The number of recovered embryos with good quality (Grade 1 + Grade 2) per AI cows was 12 ± 0.95. Moreover, no significant differences were detected in the numbers of corpora lutea (Ctrl cows, 11.8 ± 0.9 and AI cows, 14.7 ± 1.6) or circulating P4 concentration (Ctrl cows, 12.2 ± 1.5 ng/ml and AI cows, 13.8 ± 0.8 ng/ml). The iTRAQ analysis revealed a significant difference in protein content and level in the D7 UF from AI cows with multiple embryos vs. those of Ctrl cows without embryos. A total of 336 proteins were identified, of which 260 proteins were more than 2-fold higher (Figure 2A) in AI cows compared to Ctrl cows. The PCA of the 260 proteins identified 18 unique proteins in AI cows, which significantly differed from those of Ctrl cows. Additionally, the presence of embryos in the UF was positively associated with SUGT1, PPID, PDLIM1, DBI, and NMRAL1 (vectors<45°, yellow circle; Figure 2B). In contrast, the presence of embryos in the UF showed a negative association with PNP, EFHD1, and ENO1 (vectors approaching 180°, red circle; Figure 2B). The ROC curve analysis was performed to identify UF proteins that can predict the presence of embryos in the uterus on D7. The area under the ROC curve analysis (AUC) determined that SUGT1 (AUC: 0.999), PPID (AUC: 0.998), NMRAL1 (AUC: 0.995), DBI (AUC: 0.994), and PDLIM1 (AUC: 0.992) had a strong predictive power for the presence of embryos in the uterus (Figures 2C,D; Table 1).

**Table 1:** The list of parameters of factors identified by ROC analysis.

Gene ID	AUC	Optimal cutoff	Sensitivity	Specificity	PPV	NPV	PLR	NLR
SUGT1	0.999	8.4	100	100	100	100	INF	0
PPID	0.998	41.5	100	100	100	100	INF	0
NMRAL1	0.995	122.2	100	100	100	100	INF	0
DBI	0.994	4419.7	100	100	100	100	INF	0
PDLIM1	0.992	22.9	100	100	100	100	INF	0
PEBP1	0.987	1147.9	100	100	100	100	INF	0
LOC615026; PRSS1; LOC780933	0.986	145090.9	100	100	100	100	INF	0
PRDX5	0.985	801	100	100	100	100	INF	0
LDHB	0.979	3096.6	100	100	100	100	INF	0
CAPG	0.975	265.2	100	100	100	100	INF	0
PSMA1	0.974	378.1	100	100	100	100	INF	0
AKR1B1	0.965	8649.6	100	100	100	100	INF	0
TXN	0.965	1483.8	100	100	100	100	INF	0
GSTP1	0.965	774.9	100	100	100	100	INF	0
ENO1	0.96	5348.7	100	100	100	100	INF	0
PNP	0.952	5055.7	100	100	100	100	INF	0
EFHD1	0.947	346.2	100	100	100	100	INF	0
LOC100851864; CKB	0.91	4293.1	100	100	100	100	INF	0

Furthermore, GO analysis of the 260 proteins revealed significantly enriched biological process (BP), cellular component (CC), and molecular function (MF) terms, from which several neutrophil-related terms were identified in BP terms, and the most enriched CC term was “extracellular vesicular exosome” (Figure 2E).

### Proteins Identified in D7 UF Were Involved Mainly in Neutrophil-Mediated Immune Responses

**Figure 3:**
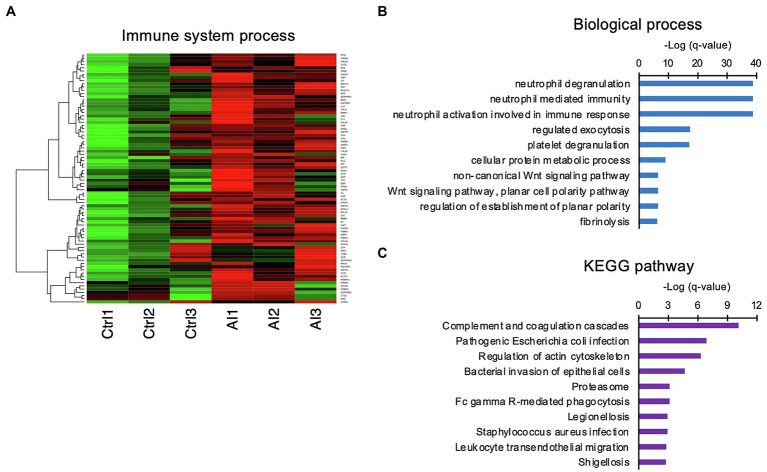
Involvement of innate immune functions in the UF obtained on D7. **(A)** Heat-map study of innate immune system-related genes in UF of inseminated (AI) and non-inseminated (Ctrl) cows. High-concentration proteins are shown in red, and low-concentration proteins are shown in green. **(B,C)** Identified immune system-related genes were functionally classified in biological GO terms **(B)** and enriched pathway analyses **(C)**.

Since the GO analysis identified “neutrophil-related terms,” the iTRAQ-identified proteins were compared with the immune-related gene database, from which a heat map was generated (Figure 3A). The matched proteins were re-evaluated with GO BP and Kyoto Encyclopedia of Genes and Genomes (KEGG) pathway analyses. The GO BP analysis identified enriched the neutrophil degranulation term, neutrophil-mediated immunity term, and neutrophil activation involved in the immune response term (Figure 3B). KEGG pathway analysis revealed enriched complement and coagulation cascade terms (Figure 3C).

### Exosomes Were Enriched in the UF-Containing Embryos on D7 Post-insemination

**Figure 4:**
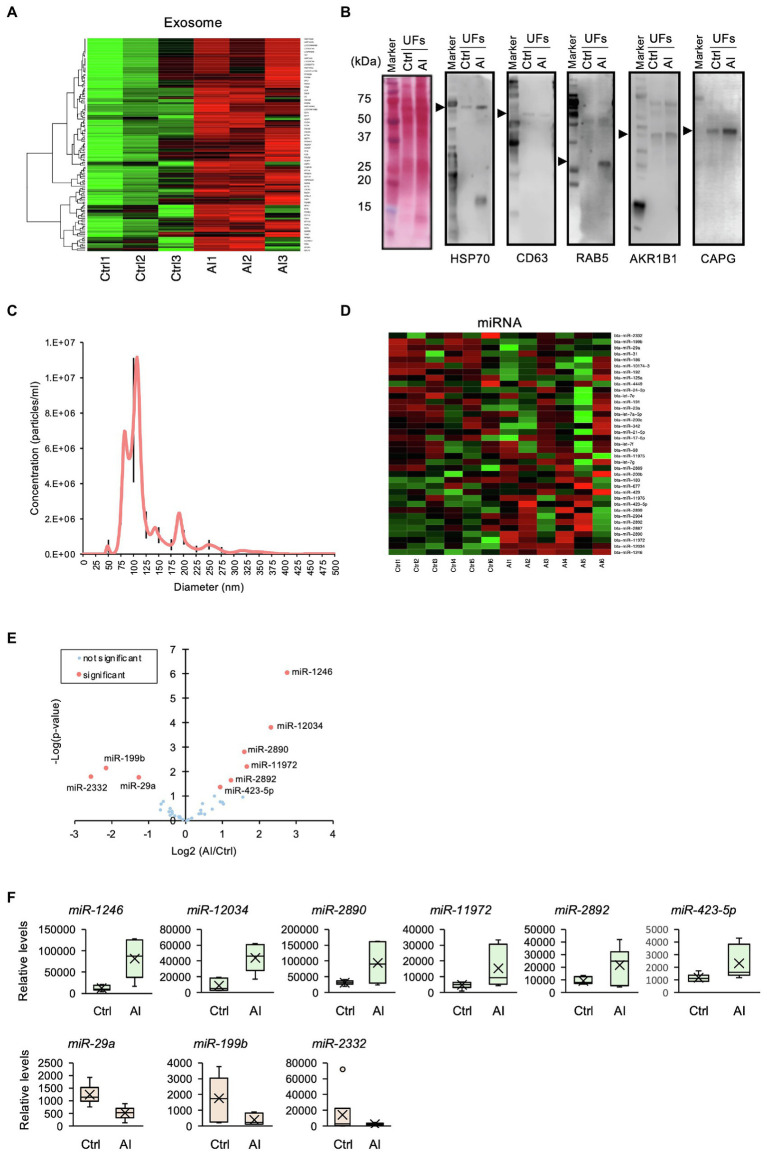
Profile of exosomal miRNAs in uterine flush (UF) collected on D7 after the estrus. **(A)** Heat-map analysis of exosome-related genes found in the UF of inseminated (AI) cows and non-inseminated (Ctrl) cows. High-concentration proteins are shown in red, and low-concentration proteins are shown in green. **(B)** The UF of Ctrl and AI cows was subjected to Western blotting, which revealed the presence of exosomal markers. The arrow heads show each target band. The polyvinylidene difluoride membrane was stained with colloidal gold. Total protein stain solution confirms total loading of protein. **(C)** Nanoparticle tracking analysis of the UF of AI cows revealed an exosomal size range of 50–150 nm. Black lines are SEM. **(D)** Heat-map analysis detected 37 exosomal miRNAs in the UF of AI and Ctrl cows. Upregulated miRNAs are shown in red, and downregulated miRNAs are shown in green. **(E)** Volcano plot showing the miRNA levels identified by miRNA-seq. The nine miRNAs highlighted in red are differentially expressed (*p* < 0.05). **(F)** Changes in identified miRNAs by the comparison between Ctrl and AI cows were determined by miRNA-seq analysis.

GO analysis identified “extracellular vesicular exosome” terms. Similar to neutrophil-related terms, we compared iTRAQ-identified proteins with the database of exosome-expressed genes and generated a heat map (Figure 4A). Using Western blot analysis, we further examined exosome markers HSP70, CD63, RAB5, AKR1B1, and CAPG. The Western blot analysis revealed exosomal markers present in UF, and the levels of these markers in UF-containing embryos were higher than those in UF from Ctrl cows (Figure 4B). These results indicate that exosomes were secreted into the uterine lumen at 7 days post-insemination. The exosomes isolated from UF in AI cows were subjected to nanoparticle tracking analysis, which showed an average size of 122.5 nm ± 35.2 nm (mean ± SEM; Figure 4C).

### Exosomal miRNAs Were Differentially Expressed in D7 UF-Containing Embryos

It was reported that exosomal miRNAs participate in dynamic changes in uterine gene expression patterns (Liang et al., 2017). To elucidate functional roles of miRNAs in exosomes, exosomal RNA isolated from D7 UF was subjected to miRNA-seq analysis, then compared with a database of miRNAs, followed by the generation of a heat map. The analysis detected 37 miRNAs in the intrauterine exosomes from Ctrl to AI cows (Figure 4D), of which three miRNAs (miR-2332, -199b, and -29a) were lowly expressed and six miRNAs (miR-1248, -12034, -2890, -11,972, -2892, and -423-5p) were highly expressed in AI vs. Ctrl cows (Figures 4E,F).

### Interaction of Exosomal miRNAs and Proteins in D7 UF-Containing Embryos

**Figure 5:**
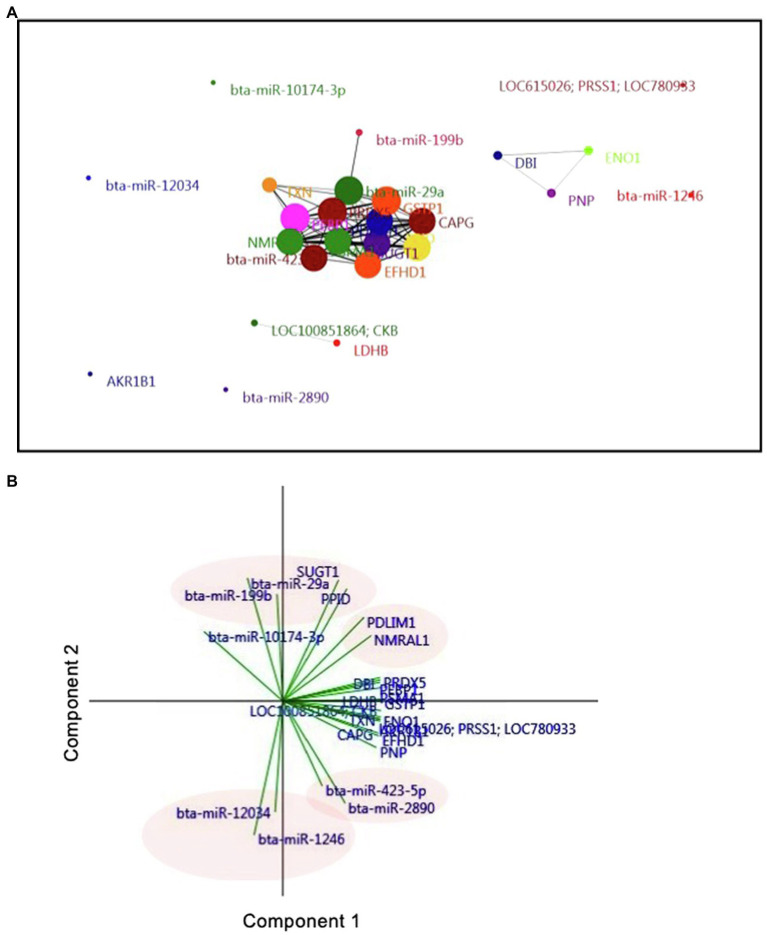
Interaction of exosomal miRNAs and proteins in D7 UF-containing embryos. **(A)** Correlation-based network analysis of exosomal miRNAs and proteins in UF. Eighteen unique proteins and nine differentially expressed miRNAs in UF of cows were mapped to a network analysis. Network analysis and visualization were carried out using the PAST and Fruchterman-Reingold algorithm as a force-directed layout algorithm. The Pearson correlation thresholds of 99% were chosen to assess the relationships between edges and nodes. Nodes are proteins and miRNAs. Edges are the interactions of all variables. The size of the nodes and edges refers to the coefficient of clustering and the coefficient of correlation, respectively. Small nodes and thin edges refer to small values. **(B)** Biplot generated from the PCA of factors, including 18 unique proteins and nine differentially expressed miRNAs found in the UF of cows. Vectors with close angles (<45°) indicate a strong correlation, perpendicular vectors indicate no correlation, and vectors in opposite directions (approaching 180°) indicate a negative correlation.

A network and PCA analyses were carried out to elucidate the relationship between miRNAs and proteins found in the UF of AI cows. To this aim, 18 proteins that significantly differed between AI cows and Ctrl cows and nine differentially expressed miRNAs were analyzed. The network analysis showed that three miRNAs, including bta-miR-29a, bta-miR-199b, and bta-miR-423-5p, were associated with many uterine proteins (Figure 5A). More precisely, the PCA showed that bta-miR-29a, bta-miR-199b, SUGT1, and PPID exhibited a strong and positive association with each other (vectors<45°, red ovals; Figure 5B). Other miRNAs, such as bta-miR-12034 and bta-miR-1246, were negatively associated with UF proteins, PDLIM1, NMRAL1, SUGT1, and PPID (vectors approaching 180°, blue oval; Figure 5B).

## Discussion

This study characterized the proteomics and exosome-derived miRNA content in UF collected from superovulated donor cows on D7 post-insemination. The proteomic analysis revealed a remarkable difference in protein content of UF in AI cows vs. Ctrl cows, and most of the identified proteins were associated with neutrophil-mediated immune responses. The expression of exosome markers was higher in UF from AI cows, and miRNA-seq identified differential expression of exosomal miRNAs in UF of AI cows vs. Ctrl cows. These findings demonstrate that the presence of D7 bovine embryos induces significant changes in uterine luminal proteins and exosome-derived miRNAs and suggest that as early as D7, a proper embryo-maternal interaction may be required to adjust the uterine environment toward the establishment of pregnancy in cattle.

One of the key immunological events that characterize the pre-hatching period of pregnancy in ruminants is the ability of the developing embryo to express antigenic MHC molecule-I on D7 post-insemination in cows (Templeton et al., 1987; Low et al., 1990). Therefore, the maternal immune system must be in place on D7 of pregnancy to prevent rejection of the semi-allogenic embryo. Indeed, recent *in vivo* investigations showed that D7 bovine embryos could modify the endometrial transcriptome and the biochemical composition of the uterine luminal fluid in the most cranial portion of the uterine horn ipsilateral to the corpus luteum (Sponchiado et al., 2017, 2019). Recently, we reported that UF collected from superovulated donor cows on D7 post-insemination induced anti-inflammatory responses and upregulation of interferon-stimulated gene expression in peripheral blood mononuclear cells *in vitro* (Rashid et al., 2018). In the present study, 336 proteins in the D7 UF, of which 260 proteins showed over a 2-fold increase in AI cows vs. Ctrl cows. Of these, many proteins were associated with “neutrophil-related” terms and “extracellular vesicular exosome” terms, suggesting that D7 bovine embryos exist in the intrauterine innate immune microenvironment.

In particular, many proteins identified in the D7 UF were related to “neutrophil degradation,” “neutrophil-mediated immunity,” and “neutrophil activation involved in immune response.” Neutrophils are the first line of the defense mechanism in the innate immune system (Tapper, 1996); thus, they might immediately respond to the presence of embryo(s) in the uterus and play key roles in modulation of the local immune cascade for acceptance or rejection of the embryo. Neutrophils could also transfer the local IFNT-signal from the uterus to the peripheral blood neutrophils. This hypothesis was supported by our previous study, demonstrating that the peripheral blood neutrophils could respond to the IFNT-signal much earlier (D5) than peripheral blood mononuclear cells (D8; Shirasuna et al., 2012). Additionally, we reported that superovulated D7 embryos generated an anti-inflammatory immune response in peripheral blood neutrophils through the upregulation of transcripts for anti-inflammatory cytokines (TGFB1 and IL10; Talukder et al., 2019). Moreover, it was found that insemination induces rapid and transient infiltrations of neutrophils into the uterine lumen for removal of bacteria, excess/dead sperm, and tissue debris, which enhances uterine clearance and subsequent embryo receptivity (Katila, 2012). The data from the current study suggest that the pre-hatching D7 bovine embryos begin to communicate with the uterus locally through the innate immune system, which might pave the way for a tolerance of the semi-allogenic embryo toward successful pregnancy in the cow. However, the definite role of neutrophils, as a component of innate immune functions of the uterus, in pregnancy establishment in cows warrants further investigations, particularly during the pre-hatching period.

This study provides the first evidence for the existence of exosomes in the lumen of the bovine uterus on D7 of pregnancy. The intrauterine exosomes are secreted from both embryo and endometrium (Burns et al., 2016), and the number of intrauterine exosomes was shown to be markedly higher in the presence of a D17 embryo in ruminants (Nakamura et al., 2016). Although the presence of intrauterine exosomes was validated in this study, the origin of exosomes (trophectoderm or endometrium) was not determined. We speculate that the higher concentration of exosomes in the UF of AI cows could be released from the endometrium in response to the zona-pellucida encapsulated embryos.

Nucleic acids, especially miRNAs, are enriched in exosomes, suggesting that exosomes can serve as means of genetic information transfer from one cell to another. It has been reported that several miRNAs in the embryo- and endometrium-derived exosomes affect the expression of adhesion- and migration-related genes in the endometrium (Kurian and Modi, 2019). In addition, enrichment analysis of the exosome-derived miRNAs indicated their involvement in several pathways necessary for embryo-endometrial crosstalk at implantation, inflammation, cell remodeling, proliferation, and angiogenesis (Ng et al., 2013; Bidarimath et al., 2017). In this study, miRNA-seq of exosomal RNA from UF revealed 37 miRNAs, of which nine miRNAs were differently expressed between AI and Ctrl cows. Of these, miR-1246, upregulated in exosomes from AI cows, targets GSK3ß and AXIN2, and inhibitors of WNT/ß-catenin signaling, which is crucial for embryo development and placentation (Chai et al., 2016; Muralimanoharan et al., 2018; Yang et al., 2019). Indeed, the enrichment analysis identified several WNT signaling-related terms in this study. It has been reported that several WNTs and WNT-related molecules are produced and secreted in the oviduct and endometrium, which regulate embryo development during early pregnancy, such as D7 (Tríbulo et al., 2018). These findings suggest that miR-1246-containing exosomes could accelerate WNT-induced early embryonic development through inhibition of GSK3ß and AXIN2. The functional impact of other deferentially expressed miRNAs on embryo-maternal interaction during the pre-hatching period in cows is unknown and requires further investigations.

The PCA analysis identified SUGT1 as a unique protein associated with the presence of embryos in the UF. Moreover, the AUC showed that SUGT1 is an indicator of embryo presence in the UF. It has been reported that SUGT1 may be involved in innate immunity through the control of inflammasome and NF-kappaB (NF-kB) activities in mammals (Mayor et al., 2007). It has also been shown that the presence of D8 bovine embryos decreased NF-kB contents in UF, adding to the thoughts on immune privilege during early embryonic development (Muñoz et al., 2012). Of note, the network analysis and PCA revealed a strong association between bta-miR-29a, bta-miR-199b, SUGT1, and PPID. It has been reported that miR-29 decreases the chemotaxis of human neutrophils through CDK2 suppression (Hsu et al., 2019), and miR-199b negatively regulates innate and adaptive immunity by suppression of IFN gamma (Ma et al., 2011). These findings suggest that D7 embryos can regulate local innate immunity in the uterine microenvironment *via* regulation of miR-29, miR-199b, and SUGT1. However, further studies are required to confirm this hypothesis.

One could argue that the superovulation model for multiple embryo production used in this study may not represent the physiological condition normally seen with a single embryo. It was reported that the maintenance of higher circulating P4 concentration by P4 supplementation during early pregnancy leads to subtle changes in a large number of genes in conceptus and endometrium, resulting in enhancing histotroph composition and contributing to advanced conceptus elongation (Forde et al., 2009; Carter et al., 2010; Forde et al., 2010). Thus, the present model used may advance the endometrial response to the embryos, i.e., the observed changes on D7 of the present study could represent the physiological changes induced by a single embryo normally seen several days later. We previously reported that the presence of multiple D7 embryos amplified the embryo-derived signals in the uterus while a single embryo did not (Talukder et al., 2017; Passaro et al., 2018). As expected, in the present study, Japanese Black cows’ responsiveness to hormone treatment was highly homogeneous, of which the number of recovered embryos with good quality per AI cows was 12 ± 0.95. Therefore, it is likely that using the model with multiple embryos in our study may have amplified the response in gene expression, rather than causing unphysiological condition or response, in the intrauterine environment.

## Conclusion

In conclusion, this study provides evidence that the presence of D7 multiple pre-hatching blastocysts induces significant changes in the relative and absolute protein and miRNA contents of UF. The comparison of global protein analysis of UF between AI and Ctrl cows revealed the involvement of innate immunity and the increase of exosomes, including several miRNAs, in the uterine lumen on D7 in the presence of multiple embryos. These findings suggest that a network of proteins and miRNA components in exosomes could contribute to the uterine microenvironment for embryo development and successful pregnancy. The impact of these proteins and miRNAs on the immunological regulation of embryo-maternal communication and the establishment of pregnancy in cattle warrants further investigations.

## Data Availability Statement

The datasets presented in this study can be found in online repositories. The names of the repository/repositories and accession number(s) can be found at https://www.ddbj.nig.ac.jp/, DRA010067.

## Ethics Statement

The animal study was reviewed and approved by the Animal Experiments Ethics Committee, Obihiro University of Agriculture and Veterinary Medicine, Japan.

## Author Contributions

Conceptualization: MS, KI, and AM. Methodology: KK, MR, RK, AT, and KN. Software, data curation, and investigation: KK. Validation: MM and AM. Formal analysis: KK and KN. Resources, supervision, project administration, and funding acquisition: AM. Writing: KK, MM, HK, and AM. Visualization: KI. All authors contributed to the article and approved the submitted version.

### Conflict of Interest

The authors declare that the research was conducted in the absence of any commercial or financial relationships that could be construed as a potential conflict of interest.

## Footnotes

^1^https://www.ddbj.nig.ac.jp/dra/index-e.html

^2^http://amp.pharm.mssm.edu/Enrichr/

^3^http://www.biosoft.hacettepe.edu.tr/easyROC/
